# Modulation of the regioselectivity of *Thermomyces lanuginosus* lipase via biocatalyst engineering for the Ethanolysis of oil in fully anhydrous medium

**DOI:** 10.1186/s12896-017-0407-9

**Published:** 2017-12-16

**Authors:** Erick Abreu Silveira, Sonia Moreno-Perez, Alessandra Basso, Simona Serban, Rita Pestana Mamede, Paulo W. Tardioli, Cristiane Sanchez Farinas, Javier Rocha-Martin, Gloria Fernandez-Lorente, Jose M. Guisan

**Affiliations:** 1 Department of Biocatalysis, Institute of Catalysis and Petrochemistry (ICP) CSIC. Campus UAM, Cantoblanco, 28049 Madrid, Spain; 2 Federal University of Sao Carlos, Sao Carlos, SP Brazil; 3 Pharmacy and Biotechnology Department, School of Biomedical Sciences, Universidad Europea de Madrid, Madrid, Spain; 4 Purolite, Unit D, Llantrisant Business Park, Llantrisant, CF72 8LF UK; 5 Embrapa Instrumentation, Sao Carlos, SP Brazil

**Keywords:** 2-monoolein, Ethyl oleate, Modulation of TLL regioselectivity, Lipase immobilization

## Abstract

**Background:**

Enzymatic ethanolysis of oils (for example, high oleic sunflower oil containing 90% of oleic acid) may yield two different reaction products depending on the regioselectivity of the immobilized lipase biocatalyst**.** Some lipase biocatalysts exhibit a 1,3-regioselectivity and they produced 2 mols of fatty acid ethyl ester plus 1 mol of sn2-monoacylglycerol (2-MAG) per mol of triglyceride without the release of glycerol. Other lipase biocatalysts are completely non-regioselective releasing 3 mols of fatty acid ethyl ester and 1 mol of glycerol per mol of triglyceride. Lipase from *Thermomyces lanuginosus* (TLL) adsorbed on hydrophobic supports is a very interesting biocatalyst for the ethanolysis of oil. Modulation of TLL regioselectivity in anhydrous medium was intended via two strategies of TLL immobilization: a. - interfacial adsorption on different hydrophobic supports and b.- interfacial adsorption on a given hydrophobic support under different experimental conditions.

**Results:**

Immobilization of TLL on supports containing divinylbenezene moieties yielded excellent 1,3-regioselective biocatalysts but immobilization of TLL on supports containing octadecyl groups yielded non-regioselective biocatalysts.

On the other hand, TLL immobilized on Purolite C18 at pH 8.5 and 30 °C in the presence of traces of CTAB yielded a biocatalyst with a perfect 1,3-regioselectivity and a very interesting activity: 2.5 μmols of oil ethanolyzed per min per gram of immobilized derivative. This activity is 10-fold higher than the one of commercial Lipozyme TL IM. Immobilization of the same enzyme on the same support, but at pH 7.0 and 25 °C, led to a biocatalyst which can hydrolyze all ester bonds in TG backbone.

**Conclusions:**

Activity and regioselectivity of TLL in anhydrous media can be easily modulated via Biocatalysis Engineering producing very active immobilized derivatives able to catalyze the ethanolysis of triolein. When the biocatalyst was 1,3-regioselective a 33% of 2-monoolein was obtained and it may be a very interesting surfactant. When biocatalyst catalyzed the ethanolysis of the 3 positions during the reaction process, a 99% of ethyl oleate was obtained and it may be a very interesting drug-solvent and surfactant. The absence of acyl migrations under identical reaction conditions is clearly observed and hence the different activities and regioselectivities seem to be due to the different catalytic properties of different derivatives of TLL.

## Background

The ethanolysis of oils catalyzed by immobilized lipases is a very relevant process: synthesis of biodiesel, synthesis of ethyl oleate, synthesis of ethyl esters of omega-3 fatty acids and so on [1–4]. Enzymatic processes may present important advantages regarding to the conventional chemical processes (e.g. esterification of free fatty acids present in oils, production of pure glycerol as sub-product, etc.) [3, 5]. However, lipases have to be immobilized and extremely stabilized in order to develop cost-effective processes [6–8].

**Fig. 1:**
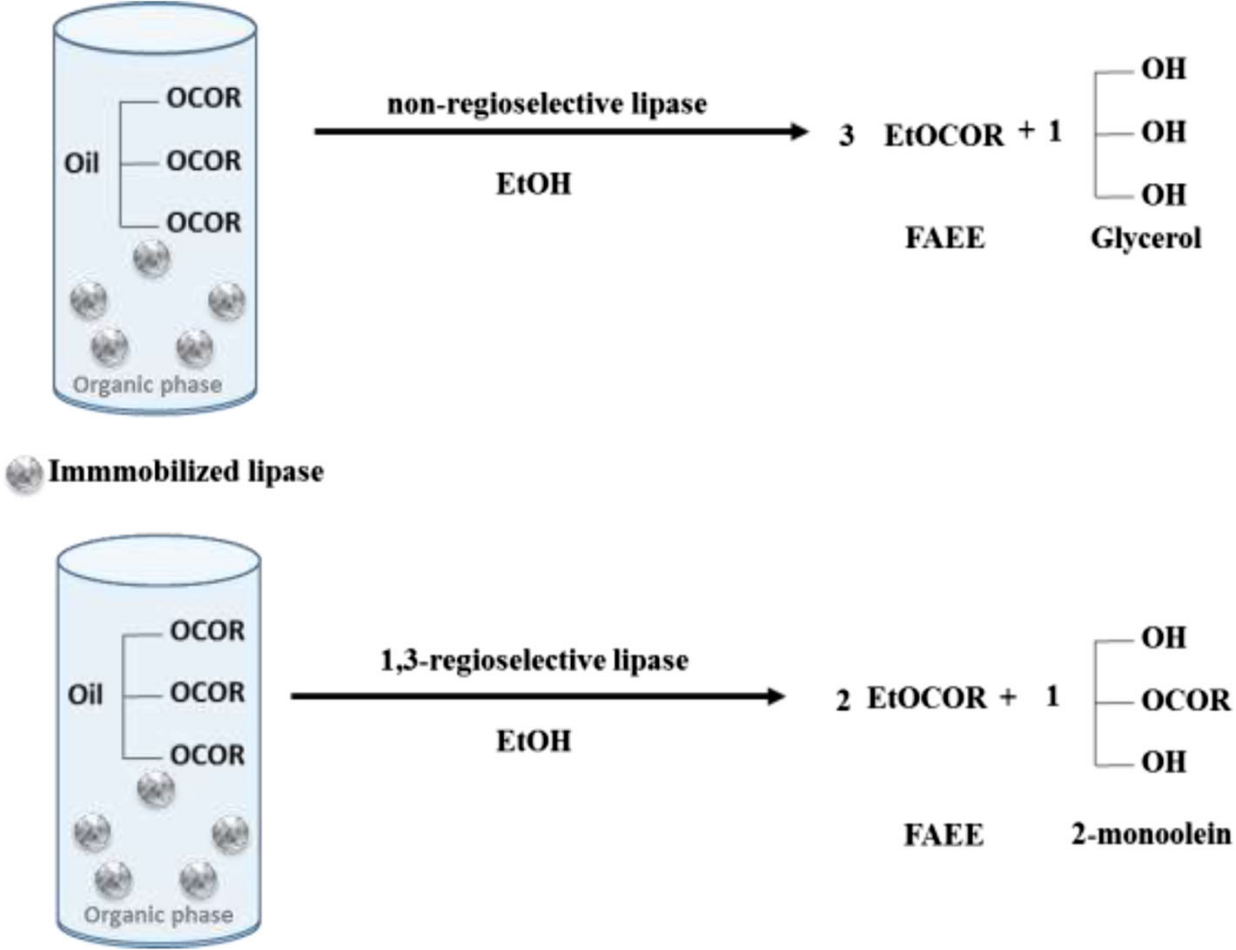
Scheme of the possible ethanolysis reactions performed by immobilized TLL depending on its selectivity

In addition to that, lipase regioselectivity may present additional advantages. On the one hand, the use of 1,3-regioselective lipases allows the transformation of 1 mol of oil and 2 mols of ethanol into 2 mols of fatty acid ethyl ester (FAEE) and 1 mol of *sn*-2-monoacylglycerol (2-MAG) with no formation of sub-products (Fig. 1). On the other hand, the use of non-selective lipases allows the quantitative conversion of 1 mol of oil and 3 mols of ethanol into 3 mols of fatty acid ethyl ester and pure glycerol as a sub-product [9] (Fig. 1). In addition, in some industrial processes, such as biodiesel and margarine fats production, non-selective lipases and acyl migration are desired [10–12]. Examples of 1,3-regioselective are lipases from *Aspergillus niger* [13], *Thermomyces lanuginosus* (TLL) [14], *Rhizopus delemar*, and *Rhizomucor mihei* [15]. Currently, only the lipase A from Candida antarctica has been reported as true sn-2-regioselective [16]. While, lipases from from *Penicillium expansum* [17], and *Pseudomonas cepacia* [18] show a random specificity.

Ethanolysis of low-cost raw triolein (high-oleic sunflower oil, HOS-oil, containing 90% of oleic acid) allows the formation of ethyl oleate or a mixture of ethyl oleate and 2-monoolein depending on lipase regioselectivity. Ethyl oleate has a number of industrial applications: e.g., in the food, cosmetic and pharmaceutical industries [19–21]. 2-monoolein is an interesting amphiphilic molecule which can be also used as an emulsifier in food, cosmetic and pharmaceutical products [22, 23]. In addition, the ethanolysis of HOS-oil can be a good model of the enzymatic synthesis of biodiesel [24].

**Fig. 2:**
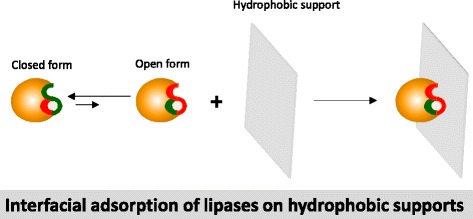
Schematic representation of the interfacial adsorption of lipases on hydrophobic supports

TLL is a 1,3-regioselective and fairly stable lipase with excellent properties to catalyze transesterification processes [1, 14]. TLL adsorbed on hydrophobic supports is a very interesting immobilized lipase biocatalyst to perform transesterification of oils [25–27]. In this case, TLL is adsorbed through the open active center fixed on the hydrophobic surface of the hydrophobic supports (Fig. 2) [28–32]. The large hydrophobic pocket surrounding the TLL active site strongly interact with the hydrophobic supports.

**Fig. 3:**
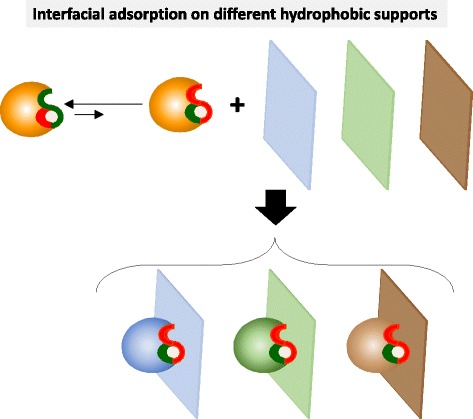
Schematic representation of the interfacial adsorption on different hydrophobic supports
- The adsorption of a given lipase on a unique hydrophobic support under different experimental conditions (pH, temperature, presence of very low concentrations of surfactants, etc.) may also promote slight changes in the exact shape of the open active center and subsequently different activity-selectivity properties of the different biocatalysts in anhydrous media (Fig. 4). Dramatic modulation of lipase properties in aqueous media under different experimental conditions have been previously reported [38–45].

In this paper, we intend the modulation of regioselectivity of TLL in anhydrous media. For this, we propose two hypotheses related to the interfacial adsorption of lipases on hydrophobic supports:

- The adsorption of a given lipase on different hydrophobic supports (different hydrophobicity, different hydrophobic moieties, etc.) may promote slightly different open forms of the adsorbed lipase and hence different catalytic properties in anhydrous media (Fig. 3). This hypothesis had been previously demonstrated for lipase reactions in aqueous medium by immobilizing lipases on different hydrophilic supports (e.g. agarose gels) activated with different hydrophobic groups (octyl, phenyl, butyl, hexyl, etc.) [33–37].

**Fig. 4:**
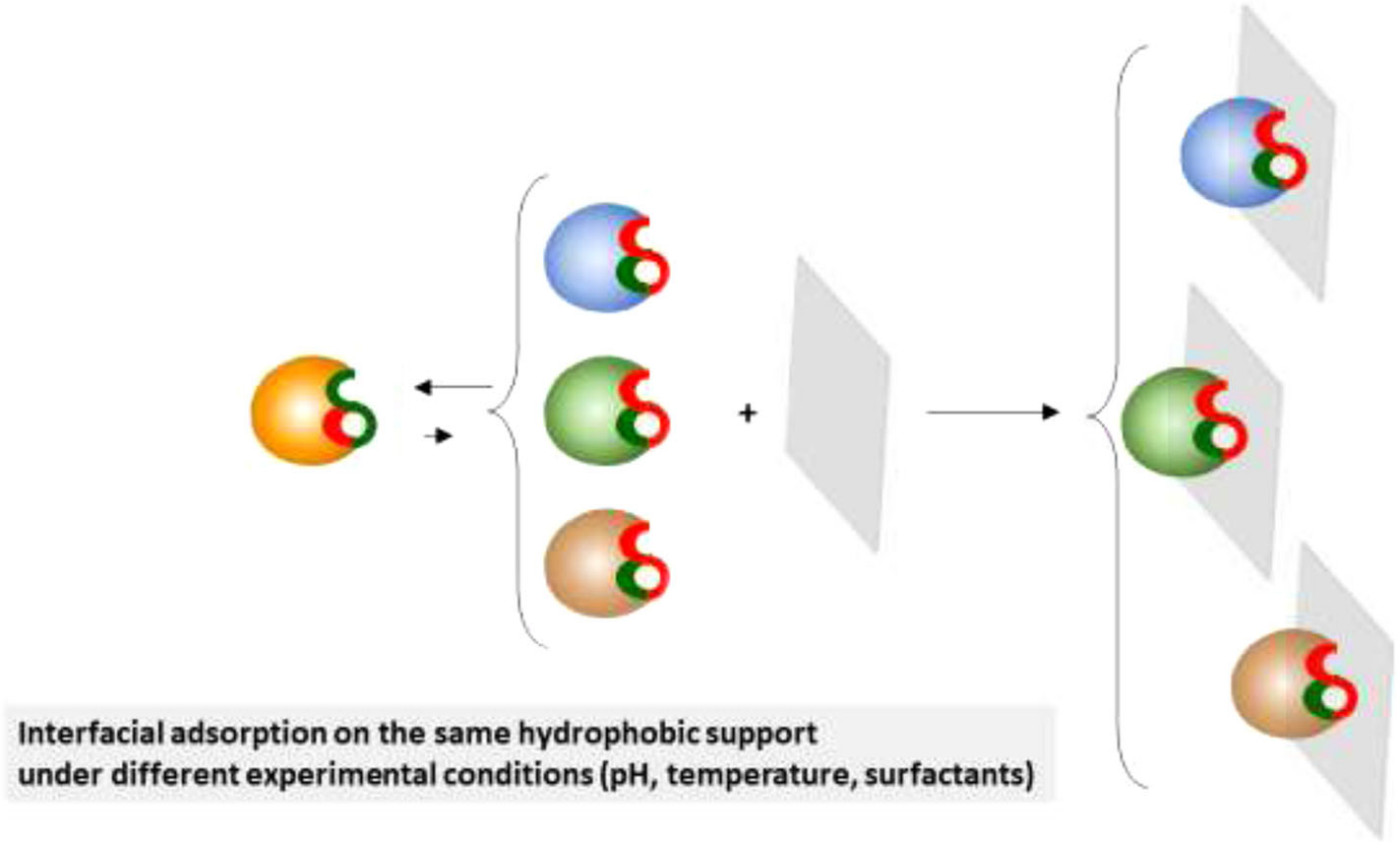
Schematic representation of the interfacial adsorption on the same hydrophobic support under different experimental conditions

From these hypothesis, our main objective is now the preparation of different immobilized TLL derivatives with different regio-selectivity (1,3-selectivity or non-selective) for the ethanolysis of HOS-oil in anhydrous media. For this purpose, TLL was immobilized, at pH 7.0 and 25 °C, on different hydrophobic supports with different hydrophobic moieties: octadecyl moieties (C18) or divinylbenezene (DVB) moieties. Thus, TLL was adsorbed on Lewatit VP OC 1600 (methacrylate porous resin containing DVB groups, Lewatit DVB), Sepabeads EC-OD (methacrylate macroporous resin containing C18 groups, Sepabeads C18), Purolite Lifetech™ ECR8806F (methacrylate macroporous resin containing C18 groups, Purolite C18) and Purolite Lifetech™ ECR1030M (methacrylate macroporous resin containing DVB groups, Purolite DVB). At present, there are few examples in the literature of the use of these Purolite supports for lipase immobilization [46–48]. While, lipases immobilized on Sepabeads C18 have been used in organic synthesis in both non-aqueous and aqueous media and biodiesel production [49–54].

On the other hand, TLL was immobilized on a unique support (Purolite C18) under different experimental conditions (pH, temperature, presence of very low concentrations of surfactant). Activity and regioselectivity of the different biocatalysts were tested by performing ethanolysis of HOS-oil by using hexane as solvent. In this way, both substrates (oil and ethanol) are fully soluble and the enzyme active site interacts with isolated substrate molecules. From a practical point of view, solvent-free ethanolysis could be advantageous but this reaction mixture is highly viscous and both substrates are immiscible. Under these interesting conditions the catalytic parameters may be influenced by additional problems (e.g. diffusion of a complex viscous mixture of oil and ethanol inside a porous structure).

## Methods

### Materials

Cetyl trimethyl ammonium bromide (CTAB), p-nitrophenyl butyrate (p-NPB), ethanol and hexane were obtained from Sigma Chemical Co. (St. Louis, Mo). High-oleic sunflower oil (HOS-oil) was purchase at a local food-shop. Lewatit VP OC 1600 (methacrylate porous resin containing divinyl benzene –DVB– groups) was purchased from Bayer (Leverkusen, Germany) (Lewatit DVB). Sepabeads EC-OD (methacrylate resin containing octadecyl –C18– groups) was obtained from Resindion S.R.L.-Mitsubishi (Milan, Italy) (Sepabeads C18). Purolite Lifetech™ ECR8806F (methacrylate macroporous resin containing octadecyl –C18– groups) (Purolite C18) and Purolite Lifetech™ ECR1030M (methacrylate macroporous resin containing divinyl benzene –DVB– groups) (Purolite DVB) were kindly donated from Purolite Ltd. (Wales, UK). Lipase from *Thermomyces lanuginosus* (Lipozyme TL 100 L) (TLL) and commercial immobilized TLL (Lipozyme TL IM) were generously donated by Novozymes (Denmark). All reagents and solvents used were of analytical or HPLC grade.

### Methods

All Experiments were made by triplicate and standard deviation was lower than 5%.

### Lipase activity assay

The hydrolytic activity of the soluble or immobilized TLL was measured as the increase in the absorbance at 348 nm (isosbestic point) produced by the release of *p*-nitrophenol in the hydrolysis of 0.4 mM pNPB in 25 mM sodium phosphate at pH 7.0 and 25 °C in a Jasco V-730 spectrophotometer with a thermostated cell and continuous magnetic stirring. The molar extinction coefficient (ε) was 5150 M^−1^ cm^−1^ under these conditions. The lipase assay was performed as the previously described increase [38]. One international activity unit (IU) was defined as μmols of hydrolyzed p-NPB per minute under the conditions described. The amount of protein was quantified using a micro BCA protein assay [55].

### Preparation of the immobilized derivatives of TLL on Purolite C18 under different experimental conditions

Immobilized derivatives were prepared under different experimental conditions: pH (5, 7 and 8), temperature (4 and 30 °C) and the absence or presence of a 0.005% of CTAB. CTAB promotes very interesting hyper-activations of soluble TLL [38]. 100 mL of enzyme solution (containing 2 mg of TLL/mL of solution) under different experimental conditions were added to 10 g of Purolite C18. Immobilization yields at a given set time were calculated by comparing the activity of a reference suspension (the enzyme under exactly the same immobilization conditions) and the activity of the supernatant under these immobilization conditions. A blank with soluble TLL under identical experimental conditions was prepared in order to check the activity of soluble TLL during the immobilization process.

Low loaded derivatives (2 mg of TLL/g of Purolite) were also prepared by using identical protocol but 100 mL of enzyme solution containing 0.2 mg of TLL/mL.

### Preparation of different immobilized derivatives of TLL using different hydrophobic supports

Soluble TLL was adsorbed on the following supports: Sepabeads C18, Purolite DVB, Lewatit DVB. The immobilization processes were performed under identical experimental conditions: 25 mM of sodium phosphate buffer, pH 7.0 and 25 °C. The experimental protocol was identical to the one described above: 100 mL of a solution containing 2 mg/mL of TLL were added to 10 g of the corresponding hydrophobic support.

### Drying of immobilized lipase derivatives

Derivatives were washed with water-acetone at different volume ratios and dried by vacuum filtration on sintered glass filters. 20 mL of several distilled water-acetone solutions were added per gram of derivative for this purpose. Firstly, they were washed with water, then, the percentage of acetone in the solution was gradually increased up to 100%. The suspensions were filtered and the solvent remaining inside the porous structure of the immobilized derivatives was allowed to fully evaporate overnight after incubation of the derivatives (inside an open thick vessel) at room temperature. Then, completely dried derivatives were obtained.

### Ethanolysis of high-oleic sunflower oil in anhydrous media

Ethanolysis was carried out using 0.1 g of fully dried derivative loaded with 20 or 1 mg protein/g support. The dried derivatives were incubated in 8.2 mL of hexane in the presence of 0.2 g of molecular sieves in order to avoid the presence of water. A volume of 0.6 mL of anhydrous ethanol (5.2 mM) and 1.2 mL of sunflower oil (0.65 mM) were added in order to start the ethanolysis reaction. The reaction was carried out at 40 °C and the reaction suspension was stirred in an orbital shaker at 150 rpm. Samples of supernatant were withdrawn at 5 min intervals (using a filter to avoid the presence of the biocatalyst) and the course of the reaction was analyzed by reversed phase high-performance liquid chromatography (RP-HPLC).

Ethanolysis were also carried out by using low loaded TLL derivatives in order to evaluate possible diffusional limitations.

### Study of the support influence on acyl migration

To study the influence of the support on 2-MAG acyl migration Sepabeads C18 solid support, Sepabeads C18 TLL derivative and inactivated Sepabeads C18 TLL were loaded in three vials. To obtain the inactivated Sepabeads C18 TLL derivative, it was incubated in 50 mM sodium phosphate buffer at 80 °C and pH 7 for 10 h. Then, the inactivated preparation was vacuum filtered.

These experiments were carried out using 0.1 g of fully dried derivative loaded with 20 mg TLL/g Sepabeads C18 or without TLL. The dried derivatives were incubated in 8.2 mL of hexane in the presence of 0.2 g of molecular sieves in order to avoid the presence of water. A volume of 0.6 mL of anhydrous ethanol (5.2 mM) and 1.2 mL of 2-monoolein (0.65 mM) were added. 2-monoolein was kept as a control. The incubation was carried out at 40 °C and the suspension was stirred in an orbital shaker at 150 rpm. Samples of supernatant were withdrawn (using a filter to avoid the presence of the biocatalyst) at set times for subsequent analysis by RP-HPLC.

### HPLC-UV analysis of ethanolysis product

The reaction products were analyzed by RP-HPLC (Spectra Physic SP 100) coupled with a UV detector set at 205 nm (Spectra Physic SP 8450) using a column Ultrabase-C18, 250 × 4.6 mm, 5 μm (Analisis Vinicos, Spain) as stationary phase and a flow-rate of 1 mL/min. The column temperature was held constant at 40 °C. All samples were dissolved in 2-propanol:hexane (5:4, *v*/v). Reservoir A contained water, reservoir B contained acetonitrile and reservoir C contained 2-propanol:hexane (5:4, v/v). A 25 min ternary gradient with two linear gradient steps was employed: 30% A + 70% B in 0 min, 100% B in 10 min, 50% B + 50% C in 20 min, followed by isocratic elution with 50% B + 50% C for the last 5 min [56]. Commercial pure standards were used to identify the product reactions. Retention times were: triolein (21.5 min), 1,2-diacyl glycerol (15.8 min), ethyl oleate (12.2 min), 1-monoolein (7.8 min) and 2-monoolein (7.5 min).

### Inactivation of immobilized TLL in anhydrous hexane

0.1 mg of each dry derivative were suspended in 8.2 mL of hexane in the presence of 0.2 g of molecular sieves in order to avoid the presence of water. 15 samples of each derivative were prepared. All samples were incubated at 40 °C. Every 12 h an ethanolysis reaction was started in one sample by adding a volume of 0.6 mL of anhydrous ethanol (5.2 mM) and 1.2 mL of sunflower oil (0.65 mM). The reaction was carried out at 40 °C and the reaction suspension was stirred in an orbital shaker at 150 rpm. Samples of supernatant were withdrawn at 10 min intervals (using a filter to avoid the presence of the biocatalyst) and the course of the reaction was analyzed by reversed phase high-performance liquid chromatography (RP-HPLC) during 6 h. The decrease of triolein in the reaction mixture was measured.

## Results

### Immobilization of TLL

Table 1 shows the characteristics of the 4 carriers selected for this study. More than 99% of the TLL offered to the hydrophobic supports was immobilized under all assayed experimental conditions in less than 4 h. The soluble TLL, which was used as control, preserved full activity after 4 h of incubation under immobilization conditions. Derivatives were loaded with the same amount of enzyme: 20 mg of TLL per gram of derivative. Some other derivatives (immobilized on Purolite C18) were also loaded with only 2 mg of TLL per gram of derivative.

### Catalytic activity of different immobilized derivatives of TLL

The catalytic activity of different immobilized derivatives adsorbed on different hydrophobic supports under identical experimental conditions was quite different as shown in Table 2. For example, the enzyme adsorbed on Sepabeads C18 was more than 2-fold more active than the enzyme immobilized on Purolite DVB.

**Table 1:** Main characteristics of the carriers selected for this work

Polymer	Matrix	Functional groups^a^	Pore volume^b^	Pore diameter^c^	Surface area^c^	Water content^d^
		mmols OD/g dry support	ml/g	Å	m^2^/g	%
PUROLITE C18	Octadecyl methacrylate	1.03	0.65	616	116	64
SEPABEADS C18^e^	Octadecyl Methacrylate	n.a	0.2–0.4	100–200	60–80	55–65
LEWATIT DVB	Methacrylate cross-linked with divinyl benzene	n.a.	0.51	324	74	60
PUROLITE DVB	Methacrylate cross-linked with divinyl benzene	1.03	0.65	616	116	64

^a^Theoretical value

^b^Measured by mercury intrusion [48]

^c^Measured by BET

^d^Measured by infrared balance [48]

^e^ Values from supplier

**Table 2:** Catalytic activity and regioselectivity of TLL immobilized on different hydrophobic supports

Supports for immobilization	Ethanolysis of oil: μmols/min /gram of biocatalyst	Regioselectivity
Sepabeads C18	0.7	No selectivity
Purolite C18	0.6	No selectivity
Lewatit DVB	0.4	1,3-regioselective
Purolite DVB	0.3	1,3-regioselective
Commercial derivative Lipozyme TL IM	0.25	No selectivity

Higher differences were found for TLL immobilized on the same support (Purolite C18) under different commercial experimental conditions (Table 3). The derivative adsorbed at pH 8.5 and 30 °C in the presence of 0.005% of CTAB exhibited a very interesting activity of 2.5 μmols of ethanolyzed oil per minute per gram of biocatalyst. This activity was 10-fold higher than the one of commercial Lipozyme TL IM and 5-fold higher than the one of a similar derivative (TLL adsorbed on the same support, Purolite C18) prepared under different experimental conditions (pH 5, 4 °C and without CTAB).

**Table 3:** Catalytic activity and regioselectivity of TLL immobilized on Purolite C18 under different experimental conditions

Immobilization conditions (pH; presence/absence CTAB; Tª)	Ethanolysis of oil: μmols/min/ gram of biocatalyst	Regioselectivity
pH 5; no CTAB; 4 °C	0.5	No selectivity
pH 7, no CTAB; 25 °C	0.6	No selectivity
pH 7; with CTAB; 25 °C	0.8	No selectivity
pH 8.5; no CTAB; 30 °C	0.8	No selectivity
pH 8.5; with CTAB; 30 °C	2.5	1,3-regioselectivity

The absence of diffusional problems was tested by analyzing the reaction rates of low loaded derivatives (1 mg of TLL /g of support). The activities were exactly 20-fold lower than ones of high load ones.

### Selectivity of different immobilized TLL biocatalysts

**Fig. 5:**
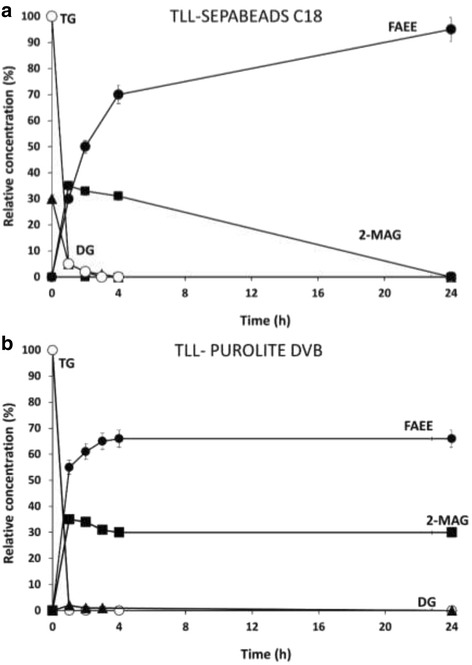
Time course of ethanolysis reaction of HOS- oil performed with TLL immobilized at 25 °C and pH 7.0 in the absence of CTAB on Sepabeads C18 (**a**) and Purolite DVB (**b**). Symbols: FAEE *(filled circle)*; TG (*unfilled circle*); 2-MAG *(filled square)*; DG *(filled triangle)*

Soluble TLL form is described as 1,3-regioselective lipase for hydrolysis of oils [14]. On the contrary, the commercial derivative Lipozyme TL IM catalyzed the hydrolysis of the 3 positions of the HOS-oil during the reaction process (Table 2). In this case, it has been demonstrated that the support (granulated silica) is the main cause of acyl migration of 2-MAG [10, 11, 57]. For example, when Lipozyme TL IM is applied for biodiesel production, the theoretical yield (66%) is exceeded, reaching a 90% yield [11]. However, ethanolysis of oil in anhydrous media was not 1,3-selective when octadecyl supports were used for TLL immobilization. These derivatives were also able to catalyze the ethanolysis of the 3 positions of HOS-oil (Table 2 and Fig. 5a). In fact, TLL immobilized on Sepabeads C18 hydrolyzed the 2-MAG quite fast and a 99% of ethyl oleate was obtained in 24 h of reaction. In contrast, when the supports contained DVB groups, 1,3-regioselectivy was perfect again (Table 2 and Fig. 5b). This result is in line with the results of Compton et al. [57].

**Fig. 6:**
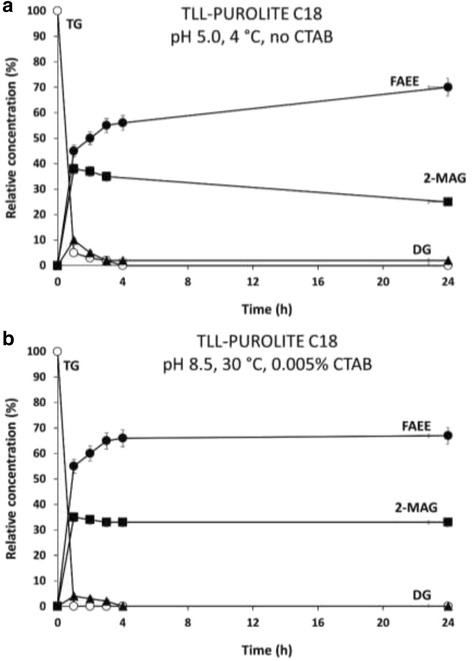
Time course of ethanolysis reaction of HOS- oil performed with TLL immobilized on Purolite C18 at pH 8.5, 30 °C with CTAB (**a**) and at pH 5.0, 4 °C without CTAB (**b**). Symbols: FAEE *(filled circle)*; TG (*unfilled circle*); 2-MAG *(filled square)*; DG *(filled triangle)*. Ethanolysis was carried out using 0.1 g of fully dried derivative (20 mg TLL/g support). A volume of 0.6 mL of anhydrous ethanol (5.2 mM) and 1.2 mL of sunflower oil (0.65 mM) were added in order to start the ethanolysis reaction. The reaction was carried out at 40 °C and the reaction suspension was stirred in an orbital shaker at 150 rpm. The course of the reaction was analyzed by RP-HPLC

On the other hand, most of the derivatives obtained in this study with Purolite C18 lost their 1,3-selectivity under anhydrous conditions. TLL immobilized at pH 5 and 4 °C in the absence of CTAB showed a rapid consumption of tryglicerides (TGs), together with the accumulation of 2-MAG and diglycerides (DG) during the early stages of the alcoholysis reaction (Fig. 6a). Due to the slow conversion of 2-MAG, the final ethyl oleate yield was lower after a 24 h period as compared to alcoholysis performed by TLL adsorbed to Sepabeads C18 (Fig. 5a). The immobilized derivative obtained by adsorption on Purolite C18 at pH 8.5 and 30 °C in the presence of CTAB exhibited a perfect 1,3-regioselectivity (Fig. 6b).

**Fig. 7:**
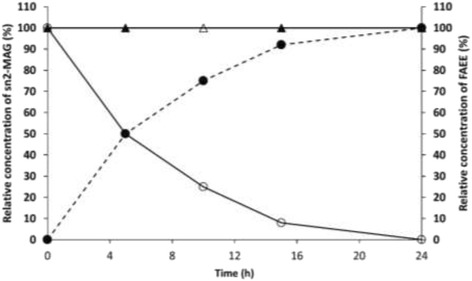
Effect of the Sepabeads C18 in the acyl migration. Symbols: FAEE production by TLL immobilized on Sepabeads C18 *(dash line and filled circle)*; 2-MAG concentration in the presence of active TLL immobilized on Sepabeads C18 *(unfilled circle)*; 2-MAG concentration in the presence of inactivated Sepabeads C18 TLL derivative *(filled triangle)*; and 2-MAG concentration in the presence of Sepabeads C18 without TLL *(unfilled triangle)*. Ethanolysis was carried out using 0.1 g of fully dried support with (20 mg TLL/g support) or without TLL. A volume of 0.6 mL of anhydrous ethanol (5.2 mM) and 1.2 mL of 2-monoolein (0.65 mM) were added in order to start the ethanolysis. The reaction was carried out at 40 °C and the reaction suspension was stirred in an orbital shaker at 150 rpm. 0.65 mM was considered as 100%. The course of the reaction was analyzed by RP-HPLC

The possible influence of the methacrylate macroporous resins on acyl migration in 2-monoolein was studied. TLL immobilized on Sepabeads C18 support was inactivated and subsequently incubated in the presence of 2-monoolein. Furthermore, the solid support (without TLL) was also incubated with 2-monoolein. In both experiments, acyl migration was not observed and 2-monoolein concentration remained constant during 24 h (Fig. 7). In addition, it was noticed a direct correlation between the increase in the FAEE concentration and the decrease in the 2-MAG concentration when the active Sepabeads C18 TLL derivative was incubated with 2-monoolein. Formation of 1-MAG was also not observed. These results are in agreement with those reported by Compton et al. [57], where more polar supports and highly acidic cation exchange resin promoted the acyl migration.

Moreover, in Fig. 6b, the concentration of 2-MAG remains unaltered in the reaction mixture (in the presence of Purolite C18) after 24 h with no migration to sn1-MAG. Thus, the decrease of 2-MAG observed in Fig. 6a (catalyzed by the same enzyme immobilized on the same supports) seems to be due the exact catalytic properties of immobilized TLL.

### Stability of immobilized TLL in anhydrous hexane at 40 °C

**Fig. 8:**
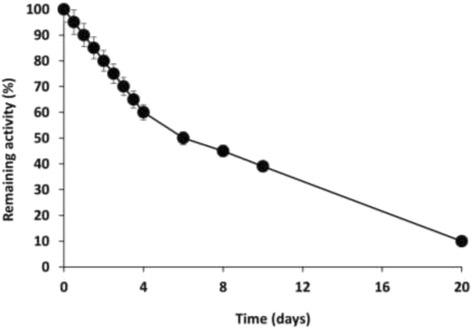
Time-course inactivation of TLL immobilized on Sepabeads C18 in anhydrous medium (hexane) at 40 °C

In Fig. 8, the time-course of inactivation of TLL adsorbed on Sepabeads C18 is shown. After 4 days, the derivative preserved 60% of its catalytic activity. The other immobilized derivative exhibited a similar stability. Stability of TLL-adsorbed on hydrophobic supports was good enough for the performance of accurate kinetic studies under these drastic experimental conditions (fully anhydrous hexane at 40 °C).

## Discussion

TLL adsorbed on different hydrophobic supports (containing DVB or C18 groups) exhibits different activity-selectivity properties for the ethanolysis of oil. These different features depend on the hydrophobic groups on the supports. The most active biocatalysts were those obtained with C18 resins and they lost the 1,3-selectivity of soluble TLL. On the other hand, the least active biocatalyst preserves the 1,3-selectivity of TLL and they are obtained with DVB resins. It seems that the adsorbed open structures of TLL may be slightly different [27, 36]. They are not depending on the resin (eg., Purolite) but they strongly depend on the hydrophobic moieties (C18 versus DVB groups).

Similar and even more relevant differences in activity-selectivity of TLL were obtained when the enzyme was adsorbed on the same hydrophobic support (Purolite C18) under different immobilization conditions (pH, temperature and presence or absence of CTAB during the immobilization process). The highest activity was obtained when the enzyme was adsorbed at 30 °C in the presence of a low concentration of surfactant (CTAB). Both conditions, higher temperature and the presence of surfactant greatly increases the catalytic activity of soluble TLL [38, 45]. In addition, CTAB has been used to increase the catalytic activity of TLL in anhydrous and low-water media by a bioimprinting strategies [58, 59]. This highly active derivative preserves the 1,3 selectivity. This most active and selective derivative (TLL immobilized on Purolite C18 at pH 8.5 and 30 °C with CTAB) could be very useful in the production of ethyl oleate and 2-monoolein in a molar ratio 2/1 [60]. Other derivatives (prepared at 4 °C) were 5-fold less active and they do not preserve 1,3-regioselectivity of TLL. Again, slight changes on the exact structure of the open molecule of TLL adsorbed on Purolite C18 may depend on the exact immobilization conditions. Based on these results, we hypothesize that some biocatalysts probably display little regioselectivity, if any, and they can hydrolyze all ester bonds in TG backbone (random specificity) depending on the immobilization conditions and nature of the support. It is also possible that these biocatalysts can show a partial hydrolysis preference for *sn*-1 and *sn*-3 positions, and after acting on these positions they can act on *sn*-2 position.

The activity-selectivity results are not affected by diffusional problems during the ethanolysis. The absence of diffusional problems in the reactions catalyzed by enzymes immobilized on porous supports can be observed by the linearity between enzyme loading in the immobilized biocatalyst and its catalytic activity [61]. In the presence of diffusional problems, there was not proportionality between observed activity and enzyme loading. In our studies, derivatives having 20 mg of TLL per gram of support was exactly 20-fold more active than derivatives having only 1 mg of TLL per gram of support. Therefore, activity and selectivity were not affected by diffusional problems. Otherwise, the crowding effect may alter activity, enzyme structure movement and substrate diffusion [62–64]. In addition, it has recently been described that the crowding effect can negatively affect the stability of the TLL immobilized on hydrophobic supports under certain conditions [65]. In our case, a crowding effect can discard due to the absence of diffusion limitations as explained above.

Previous studies have reported that solid supports can influence on 2-MAG acyl migration depending on their nature [10, 57]. Thus, this migration could be the responsible of the loss of regioselectivity of some TLL derivatives. However, when using the same support (Purolite C18) selective and non-selective biocatalyst were obtained. In these type of reactions (selective and no-selective ones) the enzyme, the support, the reaction conditions, etc., were identical and hence we can assume the absence of acyl migration in these cases. This absence of acyl migration in 2-monoolein was also demonstrated for derivatives of TLL adsorbed on Sepabeads C18 (Fig. 7). Previously, Fureby et al. [66] demonstrated that the combination of hydrophobic supports and hexane-ethanol mixtures do not facilitate acyl migrations. In addition to the support, other factors such as water activity, reaction temperature, reaction time, solvents have been proved to have important effects on the acyl migration [8].

Therefore, the different selectivities observed in the different TLL biocalysts (as well as the different catalytic activities) seem to be due to the fixation of different open forms of TLL on the different hydrophobic supports and under different experimental conditions [28, 34, 67]. It has been demonstrated that detergents can induce conformation changes in TLL structure [44]. Moreover, the effects of detergents on catalytic activity and interfacial adsorption are pH-dependent [68]. As a result, the number of the enzyme-support interactions should be very high because these changes can be fixed by the support. In contrast, if these changes were minor they would be reversible. Additional structural studies will be required to shed light on the conformational changes that occur in the enzyme.

Finally, the stability of TLL in anhydrous hexane is quite low for practical application of these interesting processes. High stabilizations of TLL adsorbed on hydrophobic agarose gels (by coating with polymers) have been reported for thermal inactivation of TLL derivatives in aqueous media [69]. Similar protocols could be tested for stabilization of immobilized TLL in anhydrous media.

## Conclusions

The immobilization of TLL on Purolite C18 under different experimental conditions allowed the preparation of immobilized derivatives with different regioselectivity and reaction rates. Immobilization at pH 8.5 and 30 °C in the presence of CTAB produced one derivative with a perfect 1,3-regioselectivity. On the contrary, immobilization at pH 5 and 4 °C in the absence of CTAB produced a non-selective derivative which catalyzed the ethanolysis of positions 1 and 3 very quickly and continuously catalyzed the hydrolysis of position 2. These results were obtained using the same support, solvent and temperature and hence migration of 2-monoolein is negligible. The non-selective TLL derivatives seem to have the effect of some conformational changes in the enzyme active center that is now able to catalyze the ethanolysis of position 2. Those supports having DVB as hydrophobic groups promoted TLL derivatives with a perfect 1,3-selectivity. On the contrary, Sepabeads C18 and Purolite C18 promoted non-selective TLL derivatives. Thus, this non-selectivity ability to hydrolyze 2-monoolein, seems to be an effect of a novel enzyme active center fixed by the support. In summary, we could exploit the same lipase for two different processes by modulating its selectivity via biocatalyst engineering: quantitative production of ethyl oleate plus glycerol or production of a 2/1 mixture of ethyl oleate and 2-monoolein. Immobilized derivatives are not very stable and this limits their industrial application. Stability of TLL-adsorbed on hydrophobic supports and incubated in anhydrous hexane is good enough for the performance of accurate kinetic studies but is quite low for practical application of these interesting processes. The increase of the stability of immobilized TLL as well as the study of ethanolysis in solvent-free systems will be the objectives of forthcoming papers.

## Abbreviations

CTAB: Cetyl trimethyl ammonium bromide
DAGs: Diacylglycerol
FAEE: Fatty acids ethyl ester
Lewatit DVB: Commercial methacrylate porous resin containing divinyl benzene –DVB– groups
Lipozyme TL IM: Commercial immobilized TLL
p-NPB: p-nitrophenyl butyrate
Purolite DVB: Commercial methacrylate resin containing DVB groups
Purolite C18: Commercial methacrylate resin containing octadecyl groups
Sepabeads C18: Commercial methacrylate resin containing octadecyl groups
2-MAG: *sn*-2-monoacylglycerol or 2-monoolein
TGs: Triglycerides
TLL: Lipase from *Thermomyces lanuginosus* or Lypozyme TL 100 L
